# Supplementary material for: Spatially Engraving Morphological Structure on a Polymeric Surface by Ion Beam Milling
Source: Polymers (Basel). 2019 Jul 23;11(7):1229. doi: 10.3390/polym11071229 (PMC6680857; doi:10.3390/polym11071229)
Supplement: Supplementary file 1 [file polymers-11-01229-s001.pdf]

Spatially Engraving Morphological Structure on a Polymeric Surface by Ion Beam Milling

Ansu Sun [1], Ding Wang [1], Honghao Zhou [1], Yifan Li [1], Chris Connor [1], Jie Kong [3,*], Jining Sun [2,*] and Ben B. Xu [1,*]

[1] Mechanical and Construction Engineering, Faculty of Engineering and Environment, Northumbria University, Newcastle upon Tyne, NE1 8ST, UK;
[2] School of Engineering and Physical Sciences, Heriot-Watt University, Edinburgh, UK;
[3] MOE Key Laboratory of Materials Physics and Chemistry in Extraordinary Conditions, Shaanxi Key Laboratory of Macromolecular Science and Technology, School of Science, Northwestern Polytechnic University, Xi'an, 710072, P. R. China.

* Correspondence: ben.xu@northumbria.ac.uk; jining.sun@hw.ac.uk; kongjie@nwpu.edu.cn;

Keywords: Ion beam milling, Topological surface, Wetting, Contact angle hysteresis

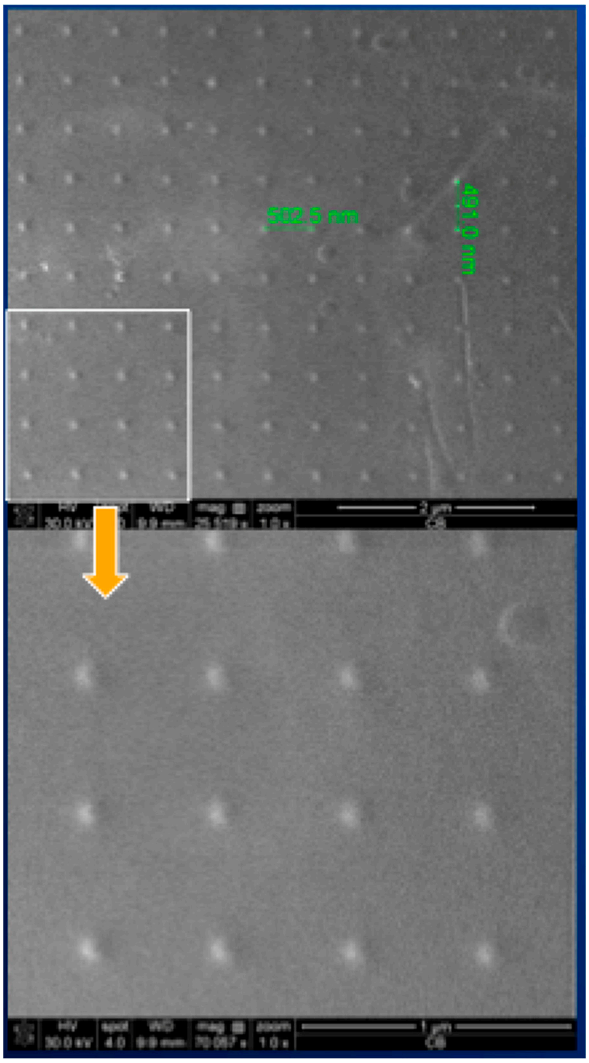

Fig S1. FIB milled nano hole array for surface wetting control
